# A Novel Automated Algorithm to Identify Lung Cancer Screening from Free Text of Radiology Orders

**DOI:** 10.1007/s11606-025-09429-2

**Published:** 2025-02-25

**Authors:** Alison S. Rustagi, Marzieh Vali, Francis J. Graham, Emily N. Lum, Christopher G. Slatore, Salomeh Keyhani

**Affiliations:** 1 Center for Data to Discovery and Delivery Innovation (3DI), San Francisco VA Health Care System, San Francisco, CA USA; 2 Division of General Internal Medicine, University of California, San Francisco, San Francisco, CA USA; 3 Northern California Institute for Research and Education, San Francisco, CA USA; 4 Center to Improve Veteran Involvement in Care, VA Portland Health Care System, Portland, OR USA; 5 National Center for Lung Cancer Screening, Veterans Health Administration, Washington, DC USA; 6 Division of Pulmonary, Critical Care, and Allergy Medicine, Oregon Health & Science University, Portland, OR USA

**Keywords:** screening, lung cancer screening, epidemiology, health services research, veterans

## Abstract

**Background:**

Lung cancer screening (LCS) is recommended for asymptomatic patients. Administrative codes for LCS may capture tests prompted by signs/symptoms.

**Objective:**

To validate an automated algorithm that identifies LCS among asymptomatic patients.

**Design:**

In this cross-sectional study, an algorithm was iteratively developed to identify outpatient low-dose chest CT scans via Current Procedural Terminology (CPT) codes, search free text of radiology orders for screening terms and signs/symptoms (e.g., cough), and classify scans as screening or not.

**Participants:**

National population-based sample of 4503 adults ages 65–80 in Veterans Health Affairs primary care, with detailed smoking history to identify LCS-eligible individuals (30 + pack-years, current tobacco use, or quit < 15 years prior).

**Main Measures:**

Algorithm specificity, sensitivity, positive predictive value (PPV), and negative predictive value (NPV) relative to manual chart review (gold standard) on 100% of screening scans and > 10% random sample of non-screening scans.

**Key Results:**

Chart review was conducted on *n* = 335 scans. The final algorithm could not classify 22% of scans, of which 73% were non-screening; these were excluded from primary analyses. Among 842 LCS-eligible individuals, the algorithm demonstrated 97% sensitivity (95%CI 91–99%) and 79% specificity (58–93%). Only 69% (61–77%) of scans classified as LCS via administrative codes were truly screening, compared to 95% of those classified as screening via the algorithm (*p* < 0.001). Algorithm performance was similar regardless of LCS eligibility, with 90% PPV (84–94%) and 93% NPV (86–97%) in the overall population regardless of tobacco cigarette history.

**Conclusions:**

An automated algorithm can accurately identify screening versus diagnostic chest imaging, a necessary step to unbiased analyses of LCS in non-randomized settings. Studies should assess the accuracy of administrative codes for LCS in other health systems.

**Supplementary Information:**

The online version contains supplementary material available at 10.1007/s11606-025-09429-2.

## INTRODUCTION

Lung cancer screening (LCS) using annual low dose computed tomography of the chest (LDCT) prevents death from lung cancer^1,2^ and is the latest cancer screening modality to be endorsed by national guidelines.^3^ Routine health data is a rich resource to understand emerging questions in LCS and has yielded early insights into adherence to routine scans,^4^ rates of procedural complications after LCS,^5,6^ stage migration associated with LCS,^7^ use of LCS by comorbidity burden,^8^ and other questions. However, these studies used administrative billing codes to measure LCS.

Diagnostic tests (e.g., those ordered for signs/symptoms such as weight loss) must be rigorously excluded to estimate harms and benefits of any screening test.^9,10^ Using administrative codes to identify LCS in routine health data may introduce bias by inadvertently capture imaging performed to evaluate signs/symptoms of disease (e.g., cough). In the National Lung Screening Trial (NLST),^1^ the landmark randomized trial that demonstrated the mortality benefit of LCS in the USA, procedures and their complications were more common in those later diagnosed with lung cancer;^1^ thus, if symptomatic individuals are counted as being “screened,” estimated rates of abnormal scans, procedures and other harms, and lung cancer detection could be biased and artificially inflated. Further, including LDCTs ordered for other indications (e.g., nodule surveillance per Fleischner criteria^11^) introduces misclassification and would overstate LCS use. These are pervasive limitations of using administrative codes to study screening.^12^ Manual chart review is the gold standard to ascertain true screening exams,^9,10,12^ but is time-intensive.

Natural language processing (NLP) has been applied to radiology reports to characterize the results of LCS,^13^ identify pulmonary nodules,^14^ and extract covariates in a cohort of patients screened for lung cancer.^15^ Text processing of Veterans Health Affairs (VHA) radiology orders has been used to accurately identify carotid imaging tests ordered to screen for carotid atherosclerosis.^16^ Whether a similar approach could identify lung imaging tests ordered to screen for lung cancer is unknown.

This study aimed to develop and validate an algorithm to automatically identify chest imaging exams done for LCS.

## METHODS

This study utilized data on chest imaging in a nationally representative, prospective cohort of adults ages 65–80 in VA primary care (*n* = 4503; NIA 5R01AG058678, PI Keyhani). Inclusion and exclusion criteria are summarized in Supplemental Fig. 1. In brief, the cohort included patients 65–80 years old with a VA primary care visit in the 2 years prior to enrollment (May 2020 to August 2023); those with dementia or functional impairment, enrolled in palliative care/hospice, residing in a nursing home, or receiving treatment for cancer (aside from localized skin cancer) were excluded. The study sample was over-weighted with cannabis users. Sampling weights were known relative to the underlying national population allowing back-calculation to generate nationally representative estimates.

All cohort members participated in a detailed, one-on-one interview at baseline that assessed lifetime tobacco use using established surveys (Supplemental Fig. 2).^17,18^ This information was used to identify those eligible for LCS under 2013 USPSTF guidelines: 30 + pack-years and current use of tobacco cigarettes or former use with quit date < 15 years prior; all cohort members were 65–80 and thus met age eligibility criteria.^19^ Our primary analysis was restricted to LCS-eligible patients. As detailed data on tobacco use may be not be known,^5,20^ and LCS occurs in practice among those who do not meet USPSTF screening criteria,^21^ we conducted sensitivity analyses including all patients regardless of smoking history.

### Automated Algorithm

**Figure 1:**
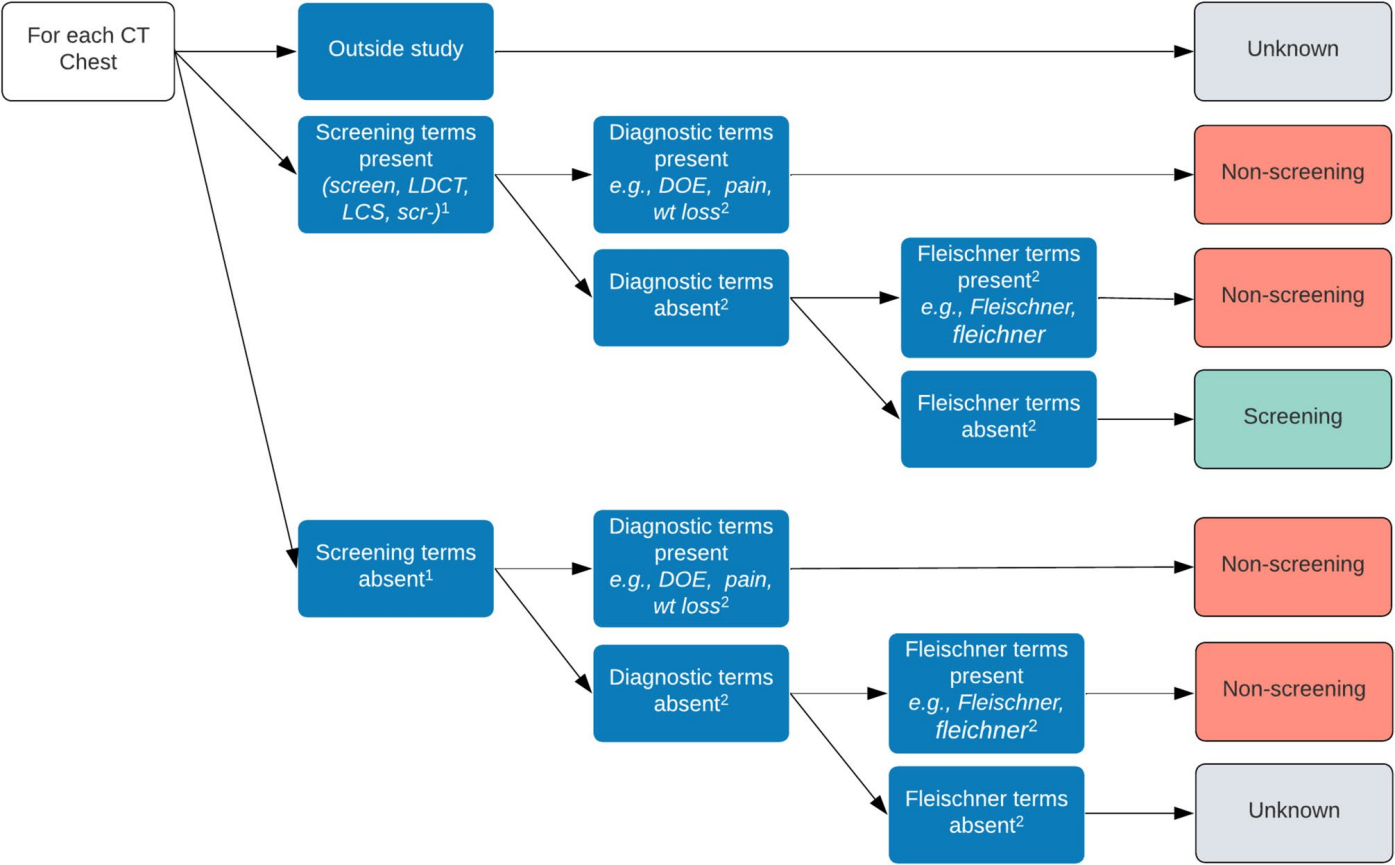
Automated algorithm to identify whether a given chest imaging study was ordered for lung cancer screening. Terms were ascertained by text search of Radiology Procedure, Study Reason, or Exam Clinical History field(s). ^1^Ignore “scr” if it appears any of these phrases: “Is this procedure to screen for malignancy?” “This study is not intended for lung cancer screening.” “HCC screening.” “Not for initial screening.” ^2^For the full list, see supplemental methods.

We iteratively developed a four-step algorithm to utilize data from administrative codes and text fields to identify screening LDCTs (Fig. 1). In VHA, each imaging study has three text-based fields. First, an imaging study is associated with standardized text-based radiology procedure code(s) such as “LDCT LUNG CANCER SCREENING”;^6,14^ these cannot be edited by a provider. Clinicians can enter free text into two fields of radiology orders: Reason for Exam and Clinical History.

First, we identified outpatient CT chest imaging by querying CPT codes for chest CT (71250, G0297, S8023, 71271) excluding those from ED or inpatient encounters (as these are typically prompted by signs/symptoms) and those imported from outside facilities as denoted by the text “Exam imported from outside” or “Imported study.” We included the CPT code for any CT chest without intravenous contrast (CPT 71250) because this code is also used for 3- and 6-month follow-up LDCTs for LCS per the American College of Radiology guidelines.^22^ We also included administrative codes specific to LCS (CPT G0297, S8023, 71271). Second, we searched free-text fields in radiology orders and radiology procedure codes for screening terms (low dose, LDCT, LCS, scr-). We cross-referenced available radiology procedure codes (e.g., LDCT LUNG CANCER SCREENING) using a published list^23^ that has been used in other LCS research.^6,24^ This ensured our list of terms comprehensively identified LCS-associated radiology procedure codes. Next, we searched free-text fields (Reason for Exam, Clinical History) for diagnostic terms reflecting signs/symptoms of disease (e.g., cough). We developed a list a priori of common presenting signs and symptoms of occult lung cancer in the ambulatory setting^25^ and iteratively expanded this list to reflect terms encountered during manual chart review. The full list of diagnostic terms is in the Supplemental Methods. Next, we searched text fields for “Fleischner” and related spellings. Fleischner guidelines are used for pulmonary nodule surveillance independent of LCS and surveillance scans that follow nodules are not considered screening.^11^ Finally, the algorithm classified scans with screening terms and without diagnostic or Fleischner terms as ordered for LCS.

### Gold-Standard Manual Chart Review

We extracted chest imaging data during the 5 years prior to cohort enrollment in October 2021. We randomly selected scans for manual chart review. As the primary goal of the algorithm is to ensure that any scan labeled as “screening” was truly done for lung cancer screening (i.e., to maximize the positive predictive value), we decided a priori to manually review 100% of scans initially classified as “screening” via the algorithm. As we iteratively updated the algorithm, we continued to review any scan newly classified as “screening.” To ensure the algorithm did not miss true screening scans, we also decided a priori to select a random sample of > 10% of those initially classified as “non-screening” via the algorithm for manual chart review. Trained chart abstractors (F.J.G., A.S.R.), one of whom is a board-certified internal medicine physician (A.S.R.), reviewed medical record notes to ascertain if a scan was non-screening (i.e., prompted by signs/symptoms of disease or for nodule surveillance per Fleischner guidelines) or screening (i.e., to detect asymptomatic lung cancer).

To ascertain the indication of a chest scan, chart reviewers searched notes in the medical record in the year prior to the scan. If the reason was not apparent from notes during that timeframe, previous notes were examined until the reason was clear. Notes from the ordering provider, the patient’s primary care provider, lung nodule tracking program coordinator, LCS program coordinator, and others were primarily used, though any note could inform this classification. If a scan was ordered due to new or worsening signs or symptoms of disease, then the scan was classified as diagnostic.

To ensure fidelity of the chart review, the two trained abstractors initially double-reviewed the same charts and discussed any discrepancies in screening classification. Abstractors proceeded independently only after 90% agreement was reached, which occurred after manual review of *n* = 41 charts. Thereafter, an ongoing random sample of 10% of scans were selected for double review to ensure fidelity. Charts with discrepant classification (e.g., classified as diagnostic via manual chart review, but screening via the algorithm) were reviewed to iteratively improve the algorithm.

### Statistical Analysis

We used standard definitions in reporting the accuracy of a diagnostic test throughout our analysis.^26^ The sensitivity of the algorithm was the proportion of all scans classified as screening via chart review that were correctly identified as screening by the algorithm; its positive predictive value (PPV) was the proportion of scans classified as screening by the algorithm that were truly screening upon chart review. The specificity of the algorithm was the proportion of all scans classified as non-screening via chart review that were correctly identified as non-screening by the algorithm; its negative predictive value (NPV) was the proportion of non-screening exams that were truly non-screening upon chart review. For the purposes of calculating algorithm sensitivity and specificity to identify LCS exams, we defined “non-screening scans” as scans ordered to evaluate signs/symptoms of disease (i.e., diagnostic) or for nodule surveillance per Fleischner guidelines (i.e., for surveillance). We calculated 95% confidence intervals (CI) of each proportion via binom.test() function in R. Scans that could not be classified by the algorithm were excluded from these calculations. In sensitivity analyses, we incorporated the complex sampling weights of the cohort into our estimates of algorithm performance (sensitivity, PPV, specificity, NPV) to assess whether the cohort sampling approach affected algorithm performance.

We calculated the PPV and NPV of administrative CPT codes commonly used to ascertain LCS relative to the gold standard of manual chart review; estimates of sensitivity and specificity of CPT codes could not be meaningfully calculated as all chest imaging studies were associated with a code. We compared the PPV/NPV of the algorithm relative to administrative codes using prop.test() function in R.

The Institutional Review Board (IRB) of the University of California-San Francisco and the Research and Development Committee at the San Francisco Veterans Affairs Health Care System approved the protocol for the present chart review study (# 21–35359) and for the underlying cohort (# 18–26608). In reporting results, we followed STROBE guidelines for cross-sectional studies.

## RESULTS

Sampled from an underlying population of 3.2 million individuals in VA primary care, the cohort included *n* = 4503 after applying inclusion and exclusion criteria (Supplemental Fig. 1). Incorporating sampling weights to generate nationally representative estimates, 82% (95%CI 81–83%) of cohort members were male, 75% (95%CI 74–76%) were white, 27% (26–29%) had a college degree, and 19% (95%CI 18–20%) were eligible for LCS at baseline (Table 1).

**Table 1:** Baseline Characteristics of the Study Cohort, a Nationally Representative Sample of Community-Dwelling Adults Utilizing VA Primary Care (*n* = 4503) and Cohort Members with ≥ 1 Chest CT Imaging Study (*n* = 1125)

	Overall cohort (*n* = 4503)		Individuals with chest imaging (*n* = 1125)^1^	
	Unweighted *n* (weighed %)	95% CI^3^	Unweighted *n* (weighted %)	95% CI^3^
Age				
65–70	1695 (38%)	36%, 39%	463 (41%)	38%, 44%
71–75	2094 (47%)	45%, 48%	489 (43%)	41%, 46%
76–80	714 (16%)	15%, 17%	173 (15%)	13%, 18%
Male	3689 (82%)	81%, 83%	887 (79%)	76%, 81%
BMI				
< 18.5	61 (1.4%)	1.1%, 1.7%	27 (2.4%)	1.7%, 3.5%
18.5–24.9	1038 (23%)	22%, 24%	319 (28%)	26%, 31%
25–29.9	1740 (39%)	37%, 40%	404 (36%)	33%, 39%
30–34.9	1064 (24%)	22%, 25%	225 (20%)	18%, 22%
35 +	593 (13%)	12%, 14%	150 (13%)	11%, 15%
Missing	7 (0.2%)	0.07%, 0.33%	0 (0%)	
Race				
White	3386 (75%)	74%, 76%	836 (74%)	72%, 77%
Black	842 (19%)	18%, 20%	234 (21%)	19%, 23%
Other	275 (6.1%)	5.4%, 6.8%	55 (4.9%)	3.8%, 6.3%
Ethnicity				
Hispanic OR Latino	213 (4.7%)	4.1%, 5.4%	46 (4.1%)	3.1%, 5.4%
Other	4290 (95%)	95%, 96%	1079 (96%)	95%, 97%
Education				
Less than high school graduate	171 (3.8%)	3.3%, 4.4%	64 (5.7%)	4.5%, 7.2%
High school/some college degree	3098 (69%)	67%, 70%	823 (73%)	70%, 76%
Bachelors and beyond	1229 (27%)	26%, 29%	238 (21%)	19%, 24%
Refused/don’t know	5 (< 0.1%)	0.03%, 0.24%	0 (0%)	
Marital status				
Married/partner	2314 (51%)	50%, 53%	503 (45%)	42%, 48%
Other	2189 (49%)	47%, 50%	622 (55%)	52%, 58%
Pack-year cigarettes				
0	1302 (29%)	28%, 30%	170 (15%)	13%, 17%
< 10	752 (17%)	16%, 18%	132 (12%)	10%, 14%
10–20	588 (13%)	12%, 14%	156 (14%)	12%, 16%
20–30	520 (12%)	11%, 13%	148 (13%)	11%, 15%
30–40	319 (7.1%)	6.4%, 7.9%	99 (8.8%)	7.3%, 11%
40–50	325 (7.2%)	6.5%, 8.0%	125 (11%)	9.4%, 13%
50 +	697 (15%)	14%, 17%	295 (26%)	24%, 29%
Tobacco smoking status				
Current	780 (17%)	16%, 18%	345 (31%)	28%, 33%
Former	2474 (55%)	53%, 56%	619 (55%)	52%, 58%
Never	1249 (28%)	26%, 29%	161 (14%)	12%, 16%
Eligible for lung cancer screening^5^				
Yes	842 (19%)	18%, 20%	378 (34%)	31%, 36%
No	3661 (81%)	80%, 82%	747 (66%)	64%, 69%
Charlson score (past 2 years), mean (SD)	4.08 (1.79)		4.61 (2.01)	
CAN score				
≤ 25	138 (3.1%)	2.6%, 3.6%	30 (2.7%)	1.9%, 3.8%
25–50	1136 (25%)	24%, 27%	203 (18%)	16%, 20%
50–75	1953 (43%)	42%, 45%	459 (41%)	38%, 44%
≥ 75	1267 (28%)	27%, 30%	433 (38%)	36%, 41%
Unknown	9 (0.09%)		0 (0%)	
Self-reported health				
Excellent	433 (9.6%)	8.8%, 11%	67 (6%)	4.7%, 7.5%
Good	2154 (48%)	46%, 49%	476 (42%)	39%, 45%
Fair	1551 (34%)	33%, 36%	462 (41%)	38%, 44%
Poor	363 (8.1%)	7.3%, 8.9%	118 (10%)	8.8%, 12%
Refused/don’t know	2 (< 0.1)	0.01%, 0.18%	2 (0.2%)	0.04%, 0.71%
Climbing stairs				
Not limited at all	2446 (54%)	53%, 56%	507 (45%)	42%, 48%
Limited a little	1367 (30%)	29%, 32%	398 (35%)	33%, 38%
Limited a lot	683 (15%)	14%, 16%	217 (19%)	17%, 22%
Refused/don’t know	7 (0.2%)	0.07%, 0.33%	3 (0.3%)	0.09%, 0.82%
Nelson eligibility				
Ineligible	516 (11%)	11%, 12%	167 (15%)	13%, 17%
Eligible	3987 (89%)	88%, 89%	958 (85%)	83%, 87%

^1^CPT codes for chest CT (71250, G0297, S8023, 71271) in the 5 years prior to cohort enrollment, excluding those from emergency department (ED) or inpatient encounters or those imported from outside facilities as denoted by the text “Exam imported from outside” or “Imported study” in the Radiology Procedure, Study Reason, or Exam Clinical History field(s)

^2^All chest imaging studies classified as “screening” via the algorithm and > 10% of those classified as “non-screening” via the algorithm were manually reviewed, excluding *n* = 41 scans that were reviewed during the initial training phase

^3^Unless otherwise indicated

^4^*p*-value from chi-squared tests for categorical variables; from *t*-tests for continuous variables

^5^30 + tobacco cigarette pack-years; current smoker or quit < 15 years prior

In the 5 years before cohort enrollment, 1125 cohort members received 1279 outpatient chest CT imaging scans conducted from August 2015 to August 2021. Chart abstractors initially reviewed 338 chest CT imaging studies: 219 of 219 scans initially classified as “screening” via the algorithm and 119 of 990 scans initially classified as “non-screening,” excluding those double-reviewed during the initial training phase (*n* = 41) and duplicates (*n* = 29). Two duplicate scans were identified after manual chart review and one scan could not be classified as screening or non-screening; these three were excluded from subsequent analyses, leaving *n* = 335 manually reviewed scans in the analysis.

Chart review occurred from March to August 2022. In total, 333 scans were associated with CPT 71250 and two were associated with CPT G0297. None was associated with S8023, which was in use during a short subset of the study period (May 2015 to October 2016). No scans were associated with CPT 71271 which was implemented in January 2021, shortly before the data pull (October 2021), and overlapped with G0297 (January 2021–December 2021). The automated algorithm could not classify 22% of scans (33 of 147 scans in the LCS-eligible group; 75 of 335 scans overall) which were excluded from further analysis. The majority of these unclassified scans were non-screening per chart review (22/33 = 66% in the LCS-eligible group; 55/75 = 73% overall). Thus, the analysis included *n* = 114 scans among LCS-eligible individuals and *n* = 260 in individuals regardless of LCS eligibility.

Among LCS-eligible individuals, the algorithm demonstrated 97% sensitivity (95%CI 91–99%) and 79% specificity (95%CI 58–93%) in identifying chest imaging ordered for LCS (Table 2). Of those scans classified as screening via the algorithm, 95% (87–98%) were truly screening upon chart review (PPV). Of those classified as non-screening, 86% (64–96%) were truly non-screening upon chart review (NPV). When applied to *n* = 260 chest imaging studies in patients regardless of LCS eligibility, the algorithm performed similarly well with 95% sensitivity, 90% PPV, 86% specificity, and 93% NPV. In sensitivity analyses, incorporating the complex sampling weights of the cohort did not meaningfully affect the estimates of algorithm performance (Supplemental Table 1). In another sensitivity analysis, we retained “unable to classify” scans in the analysis as “non-screening” scans; in the overall population regardless of tobacco cigarette history, the PPV was 90% (84–94%) and the NPV was 85% (79–90%) (Supplemental Table 2).

**Table 2:** Performance of an Automated Algorithm to Identify Lung Cancer Screening Exams in a Nationally Representative Sample of Community-Dwelling Adults Utilizing VA Primary Care

		Chart review			Total
		Screening	Non-screening		
A. Eligible for LCS*		*n* (col %, 95% CI) (row %, 95% CI)	*n* (col %, 95% CI) (row %, 95% CI)	*n*	
Algorithm	Screening	87 97% (91–99%)^†^ 95% (87–98%)^‡^	5 21% (7.9–43%) 5.4% (2.0–13%)	92	
	Non-screening	3 3.3% (0.86–10%) 14% (3.6–36%)	19 79% (58–93%)^§^ 86% (64–96%)^‖^	22	
	Total	90	24	114	
B. Overall^¶^					
Algorithm	Screening	141 95% (90–98%)^†^ 90% (84–94%)^‡^	16 14% (8.6–22%) 10% (6.1–16%)	157	
	Non-screening	7 4.7% (2.1–9.9%) 6.8% (3.0–14%)	96 86% (78–91%)^§^ 93% (86–97%)^‖^	103	
	Total	148	112	260	

Lifetime tobacco cigarette use was assessed via detailed interview at cohort enrollment. LCS eligibility was defined as follows: 30 + pack-years via tobacco cigarette smoking history and current tobacco cigarette use or former use with quit date < 15 years prior. All cohort members were 65–80 years and thus met age eligibility for LCS

*Abbreviations*: *LCS*, lung cancer screening; *VA*, Department of Veterans Affairs

^*^Excludes *n* = 33 scans that were classified as “unknown” via the final algorithm. The majority of these (22 scans or 22/33 = 66.6%) were non-screening upon chart review

^†^Sensitivity

^‡^Positive predictive value

^§^Specificity

^‖^Negative predictive value

^¶^Excludes *n* = 75 scans that were classified as “unknown” via the final algorithm. The majority of these (55 scans or 55/75 = 73.3%) were non-screening upon chart review

The algorithm misclassified few scans among LCS-eligible patients. For the *n* = 5 scans misclassified as screening, all had terms in the free-text fields that clearly indicated the ordering provider intended to order LCS (e.g., “lung cancer screening”) though chart review revealed that signs and symptoms of disease had prompted the imaging order. For the *n* = 3 scans that were misclassified as non-screening, all included terms associated with disease though documentation in the medical record indicated the primary reason for the exam was to screen for lung cancer in an asymptomatic patient.

Compared with administrative codes, the algorithm was more accurate. The PPV of administrative codes was 69% (61%, 77%), significantly lower than the algorithm (*p* < 0.001). In other words, 31% (23–39%) of outpatient chest CT scans among LCS-eligible individuals identified via administrative codes were non-screening upon chart review.

## DISCUSSION

A novel, automated algorithm utilizing administrative codes, radiology procedure codes, and free-text fields in radiology orders was 97% sensitive and 79% specific in identifying LCS imaging studies among LCS-eligible individuals, and performed similarly among all individuals regardless of LCS eligibility. Of scans classified as screening via this algorithm, 95% were truly screening upon manual chart review, the gold standard. The algorithm was more accurate than identifying LCS than administrative codes, which frequently captured non-screening scans.

Though routine data is an invaluable source of information to understand LCS, these results highlight the substantial limitations in relying on unvalidated administrative codes when ascertaining screening exams. Failure to remove diagnostic imaging studies may partially explain elevated rates of procedures observed in analyses of routine healthcare data in the pre-screening era^27^ and in the context of LCS.^5,6^ Analyses that rely on chart review^28^ may be higher quality but are also limited in terms of size by necessity; others rely on administrative codes plus Lung CT Screening Reporting & Data System (Lung-RADS) codes^29^ or certification by data entry personnel that an imaging study was conducted in the absence of signs/symptoms^30,31^ though the validity of these approaches to exclude diagnostic imaging is unknown. To advance our understanding of the impact of LCS in an unbiased way, we need standardized, rigorous methods to ascertain true screening exams among the 14.5 million US adults eligible for LCS.^32^ Otherwise, rates of LCS uptake, procedures, procedural complications, and lung cancer detection would likely be inflated by inadvertently including scans among symptomatic patients — in whom the pretest probability of disease is, on average, higher. This not only could provide false reassurance in terms of LCS utilization and ability to detect lung cancer, but also could raise false alarm in terms of procedures and complications, radiation-induced cancer, and other harms. Given that a nuanced understanding of the benefits and harms of LCS is critical to evidence-based guidelines,^33–35^ decision aids,^36^ digital tools to encourage LCS use or track uptake for targeted outreach,^37,38^ and individual patient-provider conversations via shared decision-making,^39^ accurately identifying chest CT scans performed for screening is fundamental to high-quality research on the health impacts of LCS.

The small number of scans misclassified as “screening” via the algorithm (5 of 92) could not be correctly classified even using all available data in administrative codes, radiology procedure codes, and free-text fields. These scans were ordered in the presence of signs/symptoms of disease though the ordering provider wrote free text that suggested s/he intended to screen for lung cancer. These results speak to the need to educate providers that LDCTs to screen for lung cancer are only indicated in asymptomatic patients.^3,34,35^

This study has several strengths. The automated algorithm was validated relative to manual chart review using two trained abstractors who maintained high fidelity through the chart review process; manual chart review is the gold standard but also prohibitively time-intensive to use broadly. Tobacco smoking history was assessed for all cohort members through a detailed, one-on-one interview, which allows us to rigorously identify LCS-eligible individuals. The algorithm performed similarly well in the broader cohort (including those eligible and ineligible for LCS); this suggests that the algorithm can be applied even without detailed knowledge of tobacco cigarette smoking history, which is often missing from health records.^5,20^ However, in the VA, tobacco cigarette use is systematically assessed in a widely implemented tool integrated in the electronic medical record.^38^ Further, cohort members were representative of the national population of VA primary care patients ages 65–80 and were selected with known sampling weights; using these sampling weights, we confirmed that our algorithm performed similarly well in the underlying population from which the sample was drawn. For those who opt to rely on billing codes alone, these results provide an important quantitative assessment of the (in)accuracy of billing codes. The most important metric for this algorithm is arguably not its sensitivity but rather its positive predictive value, to ensure that scans labeled “screening” are truly screening; with a positive predictive value of 90% among all individuals and 95% among LCS-eligible individuals, this algorithm exceeds the performance of more complicated natural language processing algorithms used in clinical practice.^40^

This analysis has limitations. First, we relied upon VA data which may not generalize to non-VA settings. However, VA data are uniquely important for LCS. As the nation’s largest integrated healthcare system, the VA pioneered the implementation of LCS^41^ and the systematic assessment of LCS eligibility.^38^ Veterans are at higher risk of lung cancer than the general population^42^ and more likely than their age-matched peers to be eligible for LCS.^43^ Thus, veterans represent a key population for LCS and accurately understanding how LCS is used in the VA is of particular importance. Our results also offer insights outside the VA, as the administrative codes we analyzed are broadly used in the USA. Our results suggest that relying on these codes alone may lead to biased estimates of the uptake of LCS and its impacts.

Second, our algorithm could not classify approximately one in five scans as either screening or not. We opted to exclude these “unable to classify” scans from the analysis; the majority were non-screening (66% among LCS-eligible individuals, 73% overall) and thus should be excluded from analyses of screening. Among LCS-eligible individuals, who can be identified in VA data using a standardized, systematic tool,^38^ utilizing the algorithm would result in omitting only one in ten scans that were truly screening upon chart review. We believe that this trade-off is worthwhile to minimize bias. However, to avoid introducing selection bias, other investigators might prefer to retain the “unable to classify” scans in an analysis and manually review them; with this approach, the algorithm would still eliminate approximately 80% of chart review. Another alternative would be to retain the “unable to classify” scans and treat them as “non-screening”; with this approach, the resulting PPV would be unchanged and the NPV would decrease modestly, per our sensitivity analyses.

Third, the study cohort was primarily designed to assess health impacts of cannabis and thus oversampled cannabis users; however, sampling weights were known relative to the national population of similar-aged adults in VA primary care to support inferences to the broader population. Finally, the newer billing code for LCS (CPT 71271) was not represented in our data, though we do not have reason to believe it will be used more accurately than its predecessor (G0297). The reality of day-to-day clinical practice and prior experience^44^ suggest that new and old billing codes will continue to be used inaccurately to some degree. Further, existing data on LCS using the billing codes in our analysis will continue to inform research and policy, and the American College of Radiology still requires the use of the non-LCS billing code (71250) for interval LDCTs for LCS.^22^ For these reasons, this algorithm remains useful and is an important first step to characterize the accuracy of billing codes for LCS.

In summary, an automated algorithm can accurately identify imaging to screen for lung cancer in routine VA data, a necessary first step to obtain unbiased estimates of the use and impacts of LCS in routine healthcare settings. Research is needed to validate this algorithm in non-VA health systems.

## Supplementary Information

Below is the link to the electronic supplementary material.

Supplementary file1 (DOCX 297 KB)

## Data Availability

The datasets during and/or analyzed during the current study are available from the corresponding author on reasonable request, in accordance with VA policy, and with appropriate data use agreements in place.

## Declarations

### Disclaimer

The funders had no role in the design and conduct of the study; collection, management, analysis, and interpretation of the data; preparation, review, or approval of the manuscript; or decision to submit the manuscript for publication. The opinions, findings, and conclusions presented herein represent those of the authors and not necessarily those of their employers or funders.
