# Supplementary material for: A Novel Automated Algorithm to Identify Lung Cancer Screening from Free Text of Radiology Orders
Source: J Gen Intern Med. 2025 Feb 25;40(6):1306–14. doi: 10.1007/s11606-025-09429-2 (PMC12045916; doi:10.1007/s11606-025-09429-2)
Supplement: Supplementary file 1 — Supplementary file1 (DOCX 297 KB) [file 11606_2025_9429_MOESM1_ESM.docx]

**Supplemental Table 1. Performance of an automated algorithm relative to manual chart review, incorporating complex sampling weights.**

Cohort members were selected from the underlying national population of VA primary care patients ages 65-80 with known sampling weights, which allow back-calculation of nationally representative estimates shown here. LCS eligibility was defined as: 30+ pack-years via tobacco cigarette smoking history, and current tobacco cigarette use or former use with quit date<15 years prior; all cohort members were 65-80 years and thus met age-eligibility for LCS.

|  |  | Chart review | |
| --- | --- | --- | --- |
|  |  | Screening | Non-screening |
| **A) Eligible for LCS** | | Weighted col %, 95% CI  Weighted row %, 95% CI | Weighted col %, 95% CI  Weighted row %, 95% CI |
| Algorithm | Screening | 96% (96-96%)^a^  95% (95-96%)^b^ | 17% (15-18%)  5% (4-5%) |
|  | Non-screening | 4% (4-4%)  15% (14-16%) | 83% (82-85%)^c^  85% (84-86%)^d^ |
| **B) Overall** | | | |
| Algorithm | Screening | 94% (94-95%)^a^  88% (88-88%)^b^ | 15% (15-16%)  12% (12-12%) |
|  | Non-screening | 6% (5-6%)  7% (7-8%) | 85% (84-85%)^c^  93% (92-93%)^d^ |

^a^ Sensitivity.

^b^ Positive predictive value.

^c^ Specificity.

^d^ Negative predictive value.

Abbreviations: LCS=Lung cancer screening. VA=Department of Veterans Affairs. Col=column.

**Supplemental Table 2. Performance of an automated algorithm relative to manual chart review, treating all “unable to classify” scans as non-screening.**

|  |  | Chart review | |  |  |
| --- | --- | --- | --- | --- | --- |
|  |  | Screening | Non-screening | Total |  |
| **A) Eligible for LCS** | | n  (col %, 95% CI)  (row %, 95% CI) | n  (col %, 95% CI)  (row %, 95% CI) | n |  |
| Algorithm | Screening | 87  86% (78-92%)^†^  95% (88-98%)^‡^ | 5  11% (4-24%)  5% (2-12%) | 92 |  |
|  | Non-screening* | 14  14% (8-22%)  25% (15-39%) | 41  89% (76-96%)^§^  75% (61-85%)^‖^ | 55 |  |
|  | Total | 101 | 46 | 147 |  |
| **B) Overall**^¶^ | | | | | |
| Algorithm | Screening | 141  84% (77-89%)^†^  90% (84-94%)^‡^ | 16  10% (6-15%)  10% (6-16%) | 157 |  |
|  | Non-screening^¶^ | 27  16% (11-23%)  15% (10-21%) | 151  90% (85-94%)^§^  85% (79-90%)^‖^ | 178 |  |
|  | Total | 168 | 167 | 335 |  |

^*^ Includes n=33 scans that were classified as ‘unknown’ via the final algorithm. The majority of these (22 scans or 22/33 = 66.6%) were non-screening upon chart review.

^†^ Sensitivity.

^‡^ Positive predictive value.

^§^ Specificity.

^‖^ Negative predictive value.

^¶^ Includes n=75 scans that were classified as ‘unknown’ via the final algorithm. The majority of these (55 scans or 55/75 = 73.3%) were non-screening upon chart review.

Abbreviations: LCS=Lung cancer screening. VA=Department of Veterans Affairs. Col=column.

**Supplemental Figure 1. Cohort flow diagram with detailed inclusion and exclusion criteria for the study cohort.** To enroll in the study cohort (NIA 5R01AG058678, PI Keyhani), veterans who met inclusion / exclusion criteria were sampled in a two-step method: 1) Medical records were searched for terms associated with cannabis in the prior year to identify potential cannabis users and non-users, and 2) potential cannabis users and non-users were randomly sampled at a 3:1 ratio, stratified by age. Potential cohort members were then contacted by the study team to verify eligibility, obtain informed consent, and conduct a health interview.


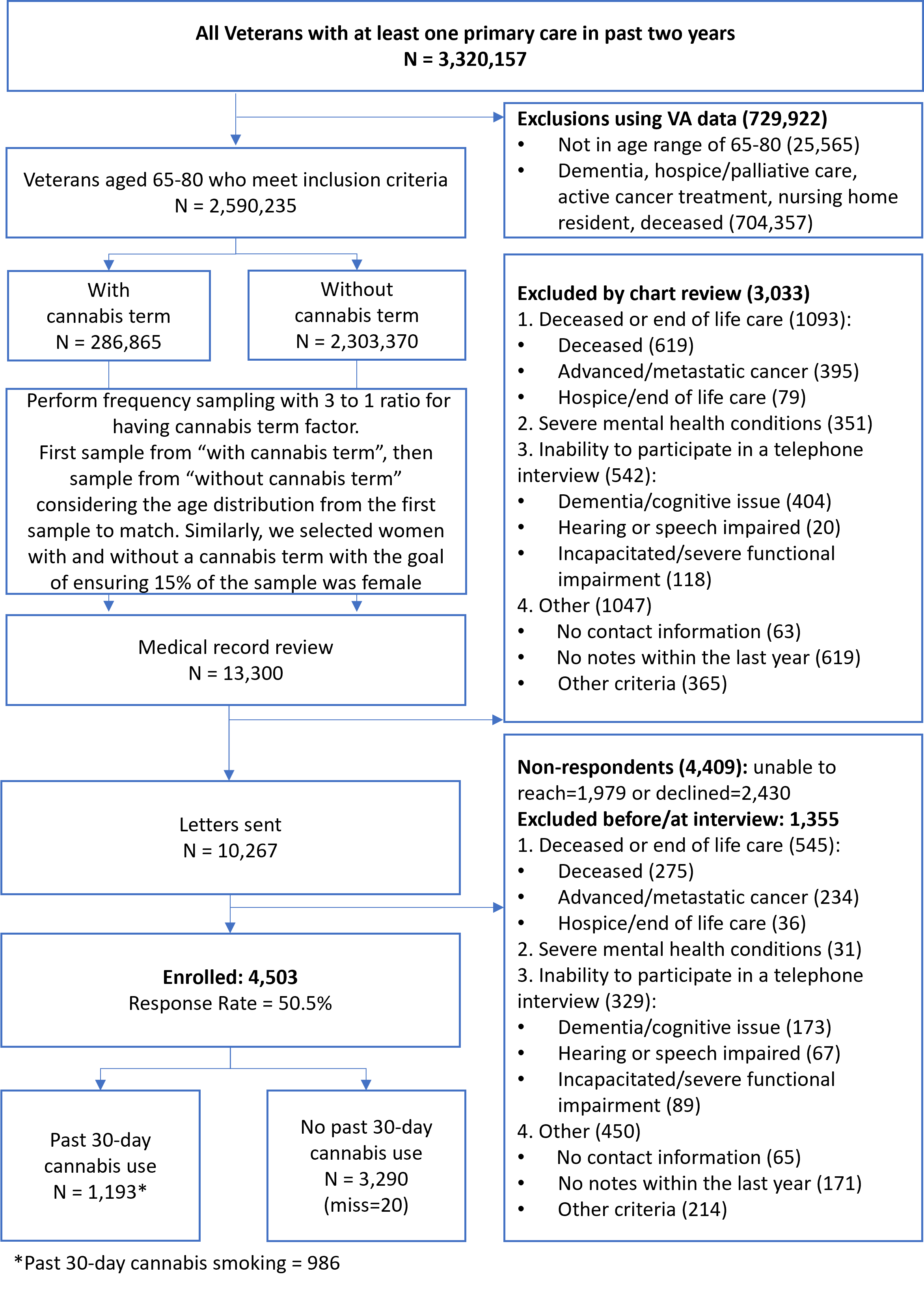


**Supplemental Figure 2. Tobacco assessment at baseline cohort interview.**

The following questions were used to assess tobacco cigarette use in a one-on-one telephone interview conducted with each cohort member at cohort enrollment.

**TOBACCO USE -- Forms**

Source: The Psychiatric Research Interview for Substance and Mental Disorders (PRISM) and NHIS

*"Now, let's talk about tobacco use."*

23. Have you ever used tobacco in any form, including vaping nicotine? q23_tb

- Yes [1]
- No [2] (skip to m1_mj)
- Refused [99] (skip to m1_mj)
- Don’t know [100] (skip to m1_mj)

*If respondent chooses [1], go to q24_tbcig*

**Cigarettes**

24. Have you smoked more than 100 cigarettes in your lifetime? q24_tbcigscreen

- Yes [1]
- No [2]
- Refused [99]
- Don’t know [100]

*If respondent chooses [1], [99], or [100] go to q25_tbcigfirst*

*If respondent chooses [2], skip to q35_tbcgl*

25. How old were you when you first smoked a cigarette? q25_tbcigfirst

- __years old [1]
- Refused [99]
- Don’t know [100]

26. When did you last smoke a cigarette? q26_tbciglast

- __days ago [1]
- __weeks ago [2]
- __months ago [3]
- __years ago [4]
- Year last used__ [5]
- Age last used__ [6]
- Refused [99]
- Don’t know [100]

*If respondent chooses [1] or [2], go to 27_tbcig30fq*

*If respondent chooses [3], [4], [5], [6], [99], or [100] go to q30_tbciglffq*

**Cigarette 30 Days**

27. In the last 30 days, how many days per week did you smoke a cigarette? q27_tbcig30fq

- Every day [1]
- 6 days/week [2]
- 5 days/week [3]
- 4 days/week [4]
- 3 days/week [5]
- 2 days/week [6]
- 1 day/week [7]
- __days/month [8]
- Refused [99]
- Don’t know [100]

28. On those days, how many cigarettes did you smoke? q28_tbcig30qt

- __cigarettes [1]
- Refused [99]
- Don’t know [100]

***[If respondent smoked more than 100 times in their lifetime…]***

**Cigarette Lifetime**

29. Have you consistently smoked that much over the entire period when you were smoking cigarettes? (interviewer note: [q28_1_tbcig30qtfill] cigarette(s) [q27_1_tbcig30fqmfill] [q27_tbcig30fq]) q29_tbciglfcont30

- Yes [1]
- No [2]
- I did not use beyond past 30 days [3]
- Refused [99]
- Don’t know [100]

*If respondent chooses [1], go to q32_tbcigquitcrt*

*If respondent chooses [2], go to q30_tbciglffq*

*If respondent chooses [3], go to q35_tbcgl*

30. Over the entire period you were smoking cigarettes, on average how often did you smoke a cigarette? q30_tbciglffq

- Every day [1]
- 6 days/week [2]
- 5 days/week [3]
- 4 days/week [4]
- 3 days/week [5]
- 2 days/week [6]
- 1 day/week [7]
- __days/month [8]
- __days/year [9]
- Refused [99]
- Don’t know [100]

31. On those days, on average how many cigarettes did you smoke? q31_tbciglfqt

- __cigarettes/day [1]
- Refused [99]
- Don’t know [100]

**For any use ever**

32. Were there any times in your life when you quit smoking cigarettes? q32_tbcigquitcrt

- Yes [1]
- No [2]
- Refused [99]
- Don’t know [100]

*If respondent chooses [2], [99], or [100], skip to q35_tbcgl*

33. Other than your final quit date, were there any other times in your life when you quit smoking cigarettes? q33_tbcigquitpst

- Yes [1]
- No [2]
- Refused [99]
- Don’t know [100]

*If respondent chooses [2], [99], or [100], skip to q35_tbcgl*

34a. In total, for about how much time did you quit smoking cigarettes? q34_a_tbcigquitdrcrt

- Quit for __days [1]
- Quit for __weeks [2]
- Quit for __months [3]
- Quit for __years [4]
- Refused [99]
- Don’t know [100]

34b. Not including the time since your final quit date, how long did you quit altogether? q34_b_tbcigquitdrpst

- Quit for __days [1]
- Quit for __weeks [2]
- Quit for __months [3]
- Quit for __years [4]
- Refused [99]
- Don’t know [100]

**Supplemental Methods.** Full list of (A) Diagnostic and (B) Fleischner terms used in the algorithm’s free text search of Radiology Procedure, Study Reason and Exam Clinical History fields.

1. Diagnostic terms: The term 'cancer' was ignored if part of the phrase "History of cancer: " as this is a prompt in some radiology order templates.

AAA, Abn chest x ray, Abn chest x-ray, abn CXR, Abnl chest x ray, Abnl chest x-ray, Abnl CXR, Abnormal chest x ray, Abnormal chest x-ray, Abnormal CXR, aneurysm, anuerysm, anorexia, aorta, aortic root, asbestos, asbestosis, atelctasis, atelectasis, biopsy, bullous emphysema, bx, CAP, Cap, cap, chest mass, chest pain, c/f CA, c/f cancer, c/f malignancy, concern for CA, concern for cancer, concern for malignancy, concerning for CA, concerning for cancer, concerning for malignancy, cough, cp, CP, Cp, COVID, covid, dyspnea, DOE, etraction, fatigue, fibrosis, f/u of nonspecific findings, granuloma, HCC screening, hemoptysis, high res, high resolution, history of cancer, h/o cancer, Hx cancer, infilt, LOA, loss of appetite, loosing weight, loss of weight, LN, lymph node, mediastinal, metastases, metastasis, mets, mucor, mucormycosis, never smoked, never smoker, night sweats, rib pain, rib, r/o cancer, rule out cancer, opacity, pain, pleural effusion, PNA, pna, Pneumonia, pneumonitis, poor appetite, opacities, opacity, shortness of breath, SOB, TAA, weight loss, WL, wt loss, zygomycosis.

1. Fleischner terms:

Fleischner, Fleishner, Fleichner, Flieschner, Fliechner, Flieshner, fleischner, fleishner, fleichner, flieschner, fliechner, flieshner.
